# Comparison of MKP and BSK-H media for the cultivation and isolation of *Borrelia burgdorferi* sensu lato

**DOI:** 10.1371/journal.pone.0171622

**Published:** 2017-02-07

**Authors:** Eva Ružić-Sabljić, Vera Maraspin, Daša Stupica, Tereza Rojko, Petra Bogovič, Franc Strle, Tjaša Cerar

**Affiliations:** 1 Institute of Microbiology and Immunology, Faculty of Medicine, University of Ljubljana, Ljubljana, Slovenia; 2 Department of Infectious Diseases, University Medical Centre Ljubljana, Ljubljana, Slovenia; University of Kentucky College of Medicine, UNITED STATES

## Abstract

The isolation of *B*. *burgdorferi* sensu lato requires the use of complex cultivation media. The aim of the study was to compare the usefulness of BSK-H (a commercial medium produced by HiMedia, India) and MKP medium. MKP and BSK-H media were prepared in accordance with the relevant protocols. *Borrelia* strains and skin culture biopsies were simultaneously inoculated into both media, incubated and checked for growth. Borrelial growth characteristics, isolation rates and characteristics of the isolated borreliae were analysed and compared. Initially, numbers of spirochaetes were higher in BSK-H than in MKP; however, in comparison with MKP, the strains subcultured in BSK-H medium were more frequently irregular, thin and non-motile, and rapidly died. In addition, the borrelial isolation rate from erythema migrans skin samples was higher in MKP than in BSK-H medium (108/171, 63.2% versus 70/171, 40.9%; p<0.0001). The far most frequently isolated species was *Borrelia afzelii* (92.9% and 97.2% strains isolated from BSK-H and MKP, respectively). Comparison of strains cultured from individual patients in both media showed differences in plasmid contents in 9/46 (19.6%) strain pairs, and protein profiles differed in 30/43 (69.8%) strain pairs, most often in the expression of OspC (in 27/28 patients OspC was expressed only in strains growing in MKP). BSK-H medium supports the growth of borrelial strains but MKP is superior with regard to the isolation rate, morphology and motility of strains. BSK-H medium supports fast initial growth of borreliae but this is followed by rapid deformation and death of the spirochaetes.

## Introduction

Lyme borreliosis is the most common tick-borne infection in Slovenia with an incidence up to 337 cases per 100,000 inhabitants [1]. Strains of the *Borrelia burgdorferi* sensu lato complex are transmitted to humans by *Ixodes* spp. ticks. Although the complex includes more than 20 genospecies, only three are commonly associated with human diseases: *Borrelia afzelii* (a dominant human pathogen in Europe), *B*. *garinii* (less frequent human pathogen in Europe) and *B*. *burgdorferi* sensu stricto (a human pathogen in North America, rare in Europe) [2, 3]. Humans acquire infection through a tick bite in a Lyme borreliosis endemic area, a natural environment supporting the life cycle of the borreliae; many species of free-living animals and birds are reservoir hosts for the spirochaetes [3, 4].

Borrelial infection can present with a wide range of clinical manifestations but most frequently as the skin lesion erythema migrans. Some other forms of clinical presentation are associated with particular *Borrelia* species: thus, acrodermatitis chronica atrophicans, a chronic skin involvement seen in Europe, is associated with *B*. *afzelii* infection, neurological disorders with *B*. *garinii* infection, and arthritis in North America with *B*. *burgdorferi* sensu stricto infection [3]. With the exception of a typical erythema migrans, diagnosis of all other clinical manifestations of borrelial infection requires laboratory confirmation [3, 5].

Two main approaches are used in laboratory diagnosis: direct methods such as culture and PCR and indirect methods based on serological tests [5–7]. Culture alone is not used routinely because of its complexity and low sensitivity. Nevertheless, although isolation of *B*. *burgdorferi* sensu lato from clinical material is a slow low-yield procedure, culture is important for the reliable diagnosis of Lyme borreliosis and for further characterization of the isolates [5–7]. To facilitate cultivation, several media have been introduced, such as modified Kelly-Pettenkofer (MKP) medium, Barbour-Stoenner-Kelly II (BSK-II) medium, and the commercially available BSK-H medium. However, these media differ in their potential to support the growth of borreliae [8–12].

The aim of the study was to compare the usefulness of MKP and BSK-H (produced by HiMedia, India) for cultivation and primary isolation of borreliae from clinical material.

## Materials and methods

The study was approved by the Medical Ethics Committee of the Ministry of Health of the Republic of Slovenia (35/08/06 and 133/06/13). The approval included skin biopsy procedure. All participants provided written informed consent to participate in the study.

### Design of the study

#### Part 1) Comparison of borrelial growth in two media

Of 183 *Borrelia*-positive MKP cultures, obtained between December 2012 and July 2013, 52 were randomly chosen for the present study. The cultures were checked for spirochaete numbers, morphology (length, thickness, curves) and motility using dark-field microscopy. Equal volumes of cultures were inoculated without delay into the MKP and BSK-H media and incubated at 33°C. After one week borrelial growth was examined and compared. Strains were subcultured in the same media and growth was examined weekly [13].

#### Part 2) Analysis of the isolation rate from clinical material in two media

Between August 2013 and January 2014, 171 adult patients diagnosed with erythema migrans at the Department of Infectious Diseases, University Medical Centre Ljubljana, Slovenia, were enrolled in the study. In each patient, two adjacent 3 mm punch skin biopsies were taken from the border of the erythema migrans lesion after first cleaning the skin with 70% alcohol and applying local anaesthesia with 2% xylocaine [14]. The samples were immediately inoculated into the MKP and BSK-H media and sent to the laboratory. Tubes were incubated at 33°C for at least nine weeks and examined weekly for the presence of spirochaetes [10, 11].

#### Preparation of the media

Ingredients of MKP and BSK-H media (HiMedia, India) are shown in S1 Table; differences in the components content and concentrations between the two media are highlighted.

MKP medium was prepared according to a previously reported protocol [10, 11, 13]. Briefly, the main components of MKP were CMRL-1066 (Gibco, No. 21-540-018) supplemented with neopeptone, HEPES buffer, citric acid, pyruvic acid, glucose, N-acetylglucosamine and sodium bicarbonate, all dissolved in sterile water and gently mixed. Gelatin (10,7 g/L), rabbit serum (55.1 mL/L) and bovine serum albumin (BSA, 9.37 g/L) were added. After mixing until the solution became clear, the medium was filtered and dispensed in glass tubes.

BSK-H (manufactured by HiMedia, India) was prepared in accordance with the manufacturer’s instructions: Part A (BSA) of the medium was suspended in sterile water, dissolved and mixed; Part B (all other components, see S1 Table) was added to part A together with a final volume of water. After obtaining a clear solution, the medium was sterilized by filtration and rabbit serum (30 mL/L) was added. After gentle mixing, the medium was filtered and dispensed in glass tubes.

Neither of the media contained antibiotics, although the manufacturer of BSK-H proposes the addition of antibiotic to its product. Possible contamination of a medium lot during preparation was checked by incubation of all tubes at 33°C for 24 hours; after that time all contaminated tubes were removed. Each medium lot was also checked for its capacity to support borrelial growth by inoculation of sample tubes with strains of *B*. *afzelii*, *B*. *garinii* or *B*. *burgdorferi* sensu stricto. After testing the quality of each medium lot (that lasted at least one week), the media were distributed to clinicians.

#### Analysis of borrelial growth

After incubation at 33°C, culture tubes were gently but thoroughly stirred and a drop of culture was examined by dark-field microscopy. At least 30 fields were examined for borrelial growth. In culture-positive tubes, the average number of spirochaetes was calculated. Borrelial morphology was defined according to spirochaete length (short or long), thickness (thin or thick) and curves (regular or irregular); borrelial motility was also determined (typical, atypical, or not motile). Short thick cells with regular curves and typical motility were interpreted as cells in “good shape” [13].

#### Analysis of isolated borrelial strains

After isolation, strains were subcultured in the same medium. Strains that grew well were identified to species level using *Mlu*I restriction fragment length polymorphism (*Mlu*I-RFLP) and pulsed-field gel electrophoresis (PFGE) as described previously [15]. Slow growing strains that did not reach a sufficiently high concentration for *Mlu*I-RFLP were identified in a polymerase chain reaction (PCR) assay based on *Mse*I-RFLP of the 5S-23S DNA amplicon [16].

For strains isolated from an erythema migrans lesion and growing sufficiently well in both MKP and BSK-H medium, plasmid and protein profiles of the two isolates were determined and compared. Plasmid profiles were obtained using PFGE, protein profiles by sodium dodecyl sulfate polyacrylamide electrophoresis (SDS-PAGE) as described previously [11, 17–20]. All paired samples for plasmid/protein profiling were prepared equally (equal concentration and equal volume per line); several, particularly those with divergent findings within individual strain pair, were electrophoresed more than once—results were consistent. In short, for protein analysis isolates were harvested in the exponential phase of growth and washed three times in PBS/Mg. After centrifugation, pallets were diluted with distilled water until cell density of 10^7^/ml, which was measured with spectrophotometer (OD_595_ 0.200). The cells were resuspended in buffer containing SDS 2.5% and 2-mercaptoethanol 2.5% and boiled for 10 min. Electrophoresis was performed in a 12% polyacrylamide gel; equal volume (30 μl) was put per line.

#### Statistics

Data are summarized with frequencies and percentages. Percentages are reported with 95% confidence intervals (CI) based on binomial distribution. Yates corrected Chi-square tests with the level of significance set at p<0.05 were used for statistical comparison of the categorical data.

## Results

Comparisons of borrelial growth in MKP and BSK-H media are reported separately for the laboratory and clinical parts of the study.

### Comparison of borrelial growth in the two media

The growth of 52 borrelial strains in MKP and BSK-H media were compared with regard to numbers of spirochaetes and their morphology and motility (Table 1). In the majority of cases the number of spirochaetes was higher in BSK-H culture tubes than in MKP; however, short thick spirochaetes with regular curves and good motility were found more frequently in MKP medium.

**Table 1:** Comparison of growth characteristics of 52 *B*. *burgdorferi* sensu lato isolates cultured in MKP and BSK-H media according to numbers of spirochaetes and their morphology and motility.

	Phenotypic parameters		
Growth characteristics	No. of analyzed *Borrelia* strains (%; 95% CI)		
	Numbers of spirochaetes	Morphology	Motility
**Comparable in MKP and BSK-H**	9 (17.3; 8.2–30.3)	8 (15.4; 6.9–28.1)	**23 (44.2; 30.5–58.7)**
**Better in MKP than in BSK-H**	6 (11.5; 4.4–23.4)	**40 (76.9; 63.2–87.5)**	**24 (46.2; 32.2–60.5)**
**Better in BSK-H than in MKP**	**37 (71.2; 56.9–82.9)**	4 (7.7; 2.1–18.5)	5 (9.6; 3.2–21.0)
**All isolates**	52 (100)	52 (100)	52 (100)

CI, confidence interval.

After subculturing strains in the same medium, the number of spirochaetes in BSK-H was again higher than in MKP medium. However, the majority of strains subcultured in BSK-H medium were irregular, non-motile, very thin and rapidly died.

### Analysis of the isolation rate from clinical material in the two media

In total, 122/171 (71.3%, 95% CI: 63.9–78.0%) erythema migrans samples were culture positive in at least one medium. For 56 (32.7%, 95% CI: 25.8–40.3%) samples, both media supported borrelial growth; in 52 (30.4%, 95% CI: 23.6–37.9%) samples, cultures were positive in MKP medium only; and in 14 (8.2%, 95% CI: 4.6–13.4%) samples, cultures were positive only in BSK-H medium (Table 2). Skin biopsies were more often culture positive in MKP than in BSK-H medium (108/171, 63.2%, 95% CI: 55.5–70.4% versus 70/171, 40.9%, 95% CI: 33.5–48.7%; p<0.0001).

**Table 2:** Isolation of *Borrelia burgdorferi* sensu lato from skin samples of erythema migrans cultured in MKP and BSK-H media.

**Medium/ culture result**			**Culture in MKP**					
			**POSITIVE**		**NEGATIVE**	**CONTAMINATED**	**ALL**	
			**Grows well (*Mlu*I-RFLP, species reported)**	**Slow growing/not cultivable (PCR)**				
**Culture in BSK-H**	**POSITIVE**	**Grows well (*Mlu*I-RFLP, species reported)**	4X **BaMla1/BaMla1**		7x **BaMla1**			
			1x **BgMlg2/BgMlg2**	∅	1x **BgMlg2**	∅	55	
			1x **BbssMlb2/BbssMlb2**		1x **BbssMlb2**			
			**(All 46)**		**(All 9)**			
		**Slow growing/not cultivable (PCR)**	8x **BaMla1/Ba**	1x **Ba/Ba**	2x **Ba**	3x **Ba** 2x CONT;		**70**
			1x **BgMlg2/Bg**			1x cont	15	
			**(All 9)**					
	**NEGATIVE**		37x **BaMla1**	2x **Ba**	36	3x CONT	**78**	
	**CONTAMINATED**		12x **BaMla1**;	1x **Ba**/1x: CONT	7	3:	**23:**	
			7x: CONT		5x CONT	3xCONT	16xCONT;	
			5x:cont		2x: cont		7xcont	
	**ALL**		104	4	**54**	**9:** 8x CONT;	**171**	
			**108**			1xcont		

*Mlu*I-RFLP, *Mlu*I-restriction fragment length polymorphism achieved with pulsed-field gel electrophoresis; PCR, polymerase chain reaction; Ba, *B*. *afzelii*; Mla1, subtype of *B*. *afzelii*; Bg, *B*. *garinii*; Mlg2, subtype of *B*. *garinii*; BbssMlb2, subtype of *B*. *burgdorferi* sensu stricto; → CONT, contamination in first tube (total 24); → cont, contamination during laboratory work (total 8).

For 95/171 (55.6%, 95% CI: 47.8–63.1%) paired skin samples results were identical: in 56 (32.7%, 95% CI: 25.8–40.3%) samples both media were culture positive, in 36 (21.1%, 95% CI: 15.2–27.9) samples both media were culture negative, and in three (1.8%, 95% CI: 0.4–5.0%) both media were contaminated (Table 2).

Contamination was found in 32/342 (9.4%, 95% CI: 6.5–13.0) skin biopsy culture tubes, more often in BSK-H than MKP medium (23/171, 13.5%, 95% CI: 8.7–19.5% versus 9/171, 5.3%, 95% CI: 2.4–9.8%; p = 0.0158). Contamination of the first (original) tube was observed in 24/342 (7.0%, 95% CI: 4.6–10.3%) samples; later, during laboratory work (subculturing, centrifuging, and microscopy), 8/342 (2.3%, 95% CI: 1.0–4.6%) tubes were found contaminated (Table 2).

### Analysis of the isolated borrelial strains

Successfully grown strains were characterized to species level using *Mlu*I-RFLP or—for slow growing strains—using *Mse*I-RFLP of the 5S-23S intergenic space amplicon. The majority of strains grew well; however, the proportion of these was higher in MKP than in BSK-H medium (104/108, 96.3%, 95% CI: 90.8–99.0 versus 55/70, 78.6%, 95% CI: 67.1–87.5%; p = 0.0005).

Although the isolation rates from the two culture media differed, no distinctions in *Borrelia* species or species subtype were found. In MKP medium, *B*. *afzelii*, *B*. *garinii* and *B*. *burgdorferi* sensu stricto were identified in 105/108 (97.2%, 95% CI: 92.1–99.4%), 2/108 (1.9%, 95% CI: 0.2–6.5%) and 1/108 (0.9%, 95% CI: 0.0–5.0) culture-positive tubes, respectively; the corresponding findings for culture-positive BSK-H medium were 65/70 (92.9%, 95% CI: 84.1–97.6%), 3/70 (4.3%, 95% CI: 0.9–12.0) and 2/70 (2.9%, 95% CI: 0.4–9.9), respectively. Overall, 117/122 (95.9%, 95% CI: 90.7–98.7%) culture-positive skin samples were infected with *B*. *afzelii*, 3/122 (2.5%, 95% CI: 0.5–7.0%) with *B*. *garinii* and 2/122 (1.6%, 95% CI: 0.2–5.8%) with *B*. *burgdorferi* sensu stricto.

All isolated strains, from MKP and/or BSK-H medium, that grew sufficiently well for *Mlu*I-RFLP analysis were subtyped: all *B*. *afzelii* strains belonged to subtype Mla1, all *B*. *garinii* strains to subtype Mlg2 and all *B*. *burgdorferi* sensu stricto strains to subtype Mlb2 (Table 2).

### Comparison of strains simultaneously isolated in MKP and BSK-H media

Fifty-six strain pairs where borreliae were isolated from skin samples in both media were compared according to species, plasmid profile and protein profile. All 56 strain pairs contained matching *Borrelia* species. Of 46 paired strains analyzed by *Mlu*I-RFLP, all pairs shared identical species subtypes: 44 strain pairs Mla1, one pair Mlg2, one pair Mlb2 (Table 2).

**Fig 1:**
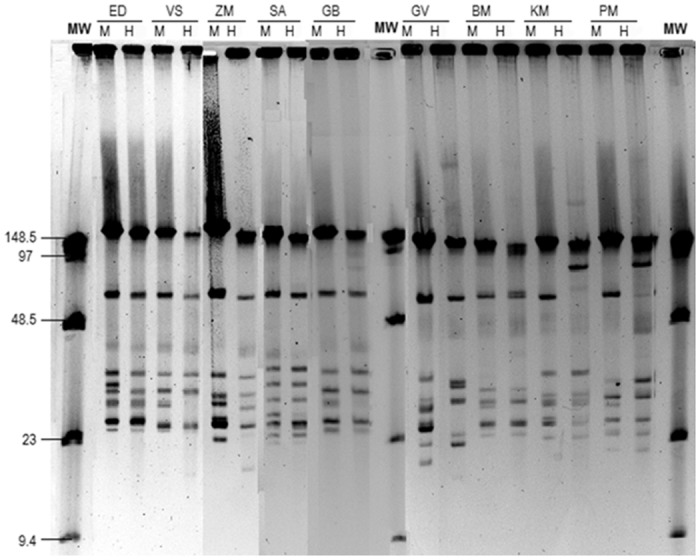
Different plasmid profiles of paired strains isolated in MKP (M) and BSK-H (H) media. MW, molecular weight marker. All isolated strains were identified as *B*. *afzelii* Mla1. Plasmid profiles of paired strains differ in the number of plasmids (significant for small plasmids under 48.5 kb) and MW (significant for large plasmids over 48.5 kb). Distinctions are evident in eight patients (ED, VS, ZM, SA, GV, BM, KM, and PM) and very likely in one patient (GB).

Plasmid profiles were successfully determined in 46/56 paired strains. Comparison of plasmid contents revealed identical plasmid profiles in 37/46 (80.4%, 95% CI: 66.1–90.6%) pairs. Nine of the 46 pairs (19.6% [95% CI: 9.4–33.9%]) differed in plasmid content (all are shown in Fig 1): two pairs (patients KM and PM) differed regarding the large plasmid (both strains from BSK-H medium had a large plasmid of about 100 kb but lacked the large plasmid of about 50 kb); in the other seven patients the paired strains differed in the content of small plasmids (plasmids under 48.5 kb) albeit in one of the patients (GB) the distinction is not clear-cut.

**Fig 2:**
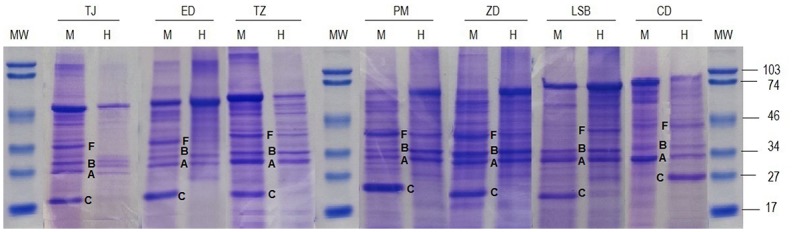
Protein profiles of paired strains isolated in MKP (M) and BSK-H (H) medium from the skin of seven patients (TJ, ED, TZ, PM, ZD, LSB, and CD). All isolated strains were identified as *B*. *afzelii* Mla1. MW, molecular weight marker; flagellin (F)–molecular weight about 41 kD; OspB (B)–molecular weight 34–36 kD; OspA (A)–molecular weight 30–33 kD, OspC (C)–molecular weight 23–27 kD.

Protein profiles were successfully determined in 43/56 paired borrelial strains, with the main interest in assessing outer surface proteins (Osps) A, B, and C and flagellin. Protein profiles differed in 30/43 (69.8%, 95% CI: 53.9–82.8) examined strain pairs (some randomly chosen strain pairs are shown in Fig 2), most often in the expression of OspC. Thus, in 28/30 patients OspC was expressed only in one of the two paired strains and in 27/28 patients the expression was shown in strains growing in MKP only while in 1/28 in BSK-H only (Fig 2, patient CD). Osps A, B, and C were differently expressed in the paired strains of one patient (1/30; not shown in Fig 2): the strain isolated from MKP lacked OspA and B, but expressed large amounts of OspC, whereas the strain isolated from BSK-H expressed Osps A and B but lacked C. In another patient (1/30; not shown in Fig 2), no Osps A and C were expressed in either media while OspB was present in the strain grown in BSK-H but not in the strain grown in MKP. Although all strains were motile in both media, deficient flagellin expression was found in 11/43 (25.6%, 95% CI: 13.5–41.2%) strain pairs: flagellin was not observed in 10 strains growing in BSK-H (Fig 2, patients TJ and ED) and in one growing in MKP (Fig 2, patient LSB). Differences in protein profiles of some randomly chosen strain pairs are shown in Fig 2.

Strain pairs of seven patients differed in both the protein (all lack OspC in BSK-H) and plasmid profiles—two of them (patients ED and PN) are shown in Figs 1 and 2.

## Discussion

Preparation of in-house medium for the culture of borreliae is an exacting and time-consuming procedure that requires trained staff and continuous quality control. The availability of good quality commercial medium is therefore an asset. The present study was designed to assess the usefulness of a commercial medium (BSK-H) for cultivation and isolation of borreliae in comparison with MPK, a medium routinely used in our laboratory. In the first part of the study we analysed borrelial growth in the two media; in the second part we used the media for isolation from clinical material. The main strengths of the study are the inclusion of a large number of patients (171) with early Lyme borreliosis and the acquisition of 122 *B*. *burgdorferi* sensu lato isolates that provided a solid basis for further analyses using several different approaches for assessment of the two media.

As shown in Table 1, comparison of the initial growth in the two media found greater number of spirochaetes in BSK-H than in MKP culture tubes, suggesting that BSK-H medium is superior in supporting rapid growth of borreliae. Although the first impression was favourable, cell deformation and death occurred soon after subculture in BSK-H. Because strains in some tubes died after only one or two passages, further characterization of the clinical isolates using *Mlu*I-RFLP identification and determination of plasmid and/or protein profiles was not achievable. Species were therefore identified using PCR assays, more often for strains growing in BSK-H than in MKP (Table 2). In contrast to BSK-H, short thick borrelial cells with regular curves were found more frequently in MKP culture tubes (Table 1), suggesting stable growth in this medium. Identical motility was found in both media in almost half of the examined strain pairs (in 44.2%) but 46.2% of strains were more motile in MKP medium (Table 1).

The growth characteristics observed in the first part of the study were similar in the clinical isolates. Nevertheless, the study corroborates previous findings indicating that growth characteristics of borreliae are influenced by the environment in which they grow [10, 11, 13].

The second part of the study encompassed 171 patients with erythema migrans whose skin biopsies were inoculated simultaneously into MKP and BSK-H media; 122/171 (71.3%) biopsies were culture positive in one or in both media (Table 2). However, in routine laboratory investigation, if culture is performed at all, only one medium is used. In the present study, positive skin biopsy results were found more often (p<0.0001) in MKP medium (108/171, 63.2%) than in BSK-H medium (70/171, 40.9%). Although the isolation rate in BSK-H medium was within the sensitivity range (40–60%) reported for culture of erythema migrans biopsies [5, 7], our study favours the use of MKP medium for routine diagnostic work.

The fastidious nature of borreliae and the changes that the organisms undergo in adaptation from living biological material to an artificial medium probably limit the isolation rate [11, 21, 22]. It appears that MKP medium is “kinder” to borreliae than BSK-H medium. However, in addition to the cultivation medium, the isolation rate is influenced by several other factors, such as the quantity of spirochaetes in a given sample (depends on the concentration of spirochaetes in a sample and on its volume), previous antibiotic treatment, local anaesthesia at the site of biopsy, transport conditions to the laboratory, and an aseptic environment for culture maintenance [11, 23]. The strains compared in our study were subjected to identical circumstances (isolation, multiplication and analysis using analogous procedures) and differed only in the culture medium; the logical conclusion, therefore, is that differences in the findings are associated with the media characteristics.

It has been reported that the concentration of borreliae in erythema migrans skin lesions is low and that they are irregularly distributed [24–26], that culture positivity of erythema migrans skin samples correlates with the burden of borreliae in the tissue and that the burden is associated with the size of the rash [14], and that the isolation rate is associated with spirochaete numbers in the sample and consequently depends upon the volume of the skin specimen [23, 27]. In the present study two adjacent 3 mm punch skin biopsies were taken from the border of the skin lesion of each patient. Although we cannot be certain that the two samples from an individual patient were of exactly the same size, and that the spirochaetes were equally distributed within each sample, it is highly unlikely that the majority of smaller specimens and/or specimens with low numbers of borreliae would all have been allocated to BSK-H medium.

The vast majority of patients with culture-positive biopsies were infected with *B*. *afzelii* (95.9%) and only few with *B*. *garinii* (2.5%) or *B*. *burgdorferi* sensu stricto (1.6%). Although the isolation rates from MKP and BSK-H medium differed, no distinctions in *Borrelia* species or species subtype were found when comparing strain pairs obtained from individual patients and isolated from the two media.

However, differences in strain pairs were evident in plasmid content (19.6% strain pairs) and more often in protein profiles (69.8% strain pairs). Previous studies reported variability in plasmid content between different *Borrelia* species and within the strains of an individual species [11, 17, 19, 28]; the latter was also found for *B*. *afzelii* strains in the present study (Fig 1). Borrelial plasmids are regarded as stable genetic elements containing important borrelial genes [29]. In general, plasmid content does not change easily and may be used as a tool for borrelial delineation within the species; the approach is particularly useful for *B*. *afzelii*, a dominant species in Slovenia. Regarding the plasmid contents of paired strains in our study, strains isolated in MKP medium possessed higher numbers of small plasmids than the corresponding strains isolated in BSK-H medium; however, two strains cultured and isolated in BSK-H contained a large plasmid of about 100 kb (plasmid dimer) but lacked the large plasmid of about 50 kb. These two strains were the only ones having a plasmid dimer in the present study. A plasmid dimer is defined as a large plasmid with a replication error [30], thus it appears that growth in BSK-H medium induces the emergence of plasmid dimers and the loss of small plasmids in some strains. However, dimerization of the 50 kb primer only happened in 2 of 46 samples/strains: patients KM and PM, H strains in Fig 1 (in patient PM dimerization was probably not complete).

Regarding protein profiles, differences between the strain pairs were even more evident (30/43, 69.8%). Strains growing in MKP expressed OspC and flagellin more often than strains growing in BSK-H. This observation supports the concept that protein expression is influenced not only by the inherent characteristics of the borreliae but also by the environment in which the spirochaetes are growing [13, 20] and goes well in parallel with earlier publications that examined BSK-H and also found some aberrant (growth) characteristics [31], including a finding indicating that Erp proteins synthesis is regulated by *B*. *burgdorferi* in response to a chemical signal(s) from the medium in addition to a temperature signal [31, 32]. However, the precise media components and specific borrelial characteristics that influence protein expression as a response to environmental conditions are unknown. Our study emphasizes the influence of the growth environment on borrelial plasmid content and even more markedly on the protein content, but does not provide an exact explanation(s) for the superiority/inferiority of either medium tested. We propose that differences in concentrations of one or several medium components (glucose, BSA, rabbit serum), differing sources of BSA, rabbit serum and even water, disparities between yeast extract versus gelatine, may all influence the distinctions between the two media. The answers to these questions might have a substantial impact on the development of new commercial media for the culture of borreliae.

## Conclusion

BSK-H medium supports the growth of borrelial strains but MKP medium appears advantageous regarding the isolation rate, and morphology and motility of strains. BSK-H medium supports rapid initial borrelial growth but this is followed by cell deformation and death. The results of the study corroborate previous findings indicating that the growth characteristics of borreliae are influenced by the environment in which they grow.

## Supporting information

S1 Table Components of MKP and BSK-H media for growth of *Borrelia burgdorferi* sensu lato; differences between the media are highlighted (in green). (DOCX)
