# Supplementary material for: Comparison of MKP and BSK-H media for the cultivation and isolation of Borrelia burgdorferi sensu lato
Source: PLoS One. 2017 Feb 7;12(2):e0171622. doi: 10.1371/journal.pone.0171622 (PMC5295711; doi:10.1371/journal.pone.0171622)
Supplement: S1 Table — (DOCX) [file pone.0171622.s001.docx]

**S1 Table.** Components of MKP and BSK-H media for growth of *Borrelia burgdorferi* sensu lato; differences between the media are highlighted (in green).

| **Components** | **BSK-H medium**  **(g/L)** | **MKP medium**  **(g/L)** |
| --- | --- | --- |
| L-Alanine | 0.025 | 0.025 |
| L-Arginine | 0.05787 | 0.07 |
| L-Aspartic Acid | 0.03 | 0.03 |
| L-Cysteine·HCl·H2O | 0.26 | 0.260 |
| L-Cystine | 0.02 | 0.026 |
| L-Glutamic acid | 0.075 | 0.075 |
| Glycine | 0.05 | 0.05 |
| L-Histidine HCl·H2O | 0.02 | 0.02 |
| Trans-4-hydroxy-L-proline | 0.01 | 0.01 |
| L-Isoleucine | 0.02 | 0.02 |
| L-Leucine | 0.06 | 0.06 |
| L-Lysine·HCl | 0.07 | 0.07 |
| L-Methionine | 0.015 | 0.015 |
| L-Phenylalanine | 0.025 | 0.025 |
| L-Proline | 0.04 | 0.04 |
| L-Serine | 0.025 | 0.025 |
| L-Threonine | 0.03 | 0.03 |
| L-Tryptophan | 0.01 | 0.01 |
| L-Tyrosine | 0.04 | 0.058 |
| L-Valine | 0.025 | 0.025 |
| N-Acetyl-D-glucosamine | 0.4 | 0.4 |
| L-Ascorbic acid | 0.05 | 0.05 |
| PABA | 0.00005 | 0.00005 |
| D-Biotin | 0.00001 | 0.00001 |
| Choline chloride | 0.0005 | 0.0005 |
| Citric acid·3Na·2H2O | 0.7 | 0.7 |
| Coenzyme A | 0.0025 | 0.0025 |
| Cocarboxylase | 0.001 | 0.001 |
| 2'-Deoxyadenosine | 0.01 | 0.01 |
| 2'-Deoxyguanosine | 0.01 | 0.01 |
| 2'-Deoxycytidine·HCl | 0.0116 | 0.01 |
| Flavin adenine dinucleotide·2Na | 0.000106 | 0.001 |
| Folic acid | 0.00001 | 0.00001 |
| Myo-inositol | 0.00005 | 0.00005 |
| 5-Methyldeoxycytidine | 0.0001 | 0.0001 |
| β-NAD | 0.007 | 0.007 |
| β-NADP·Na | 0.001 | 0.001 |
| Niacinamide | 0.000025 | 0.00002 |
| Nicotinic acid | 0.000025 | 0.00002 |
| D-Pantothenic acid·hemicalcium | 0.00001 | - |
| Pyridoxal·HCl | 0.000025 | 0.00002 |
| Pyridoxine·HCl | 0.000025 | 0.00002 |
| Pyruvic acid·Na | 0.8 | 0.8 |
| Riboflavin | 0.00001 | 0.00001 |
| Thiamine·HCl | 0.00001 | 0.00001 |
| Thymidine | 0.01 | 0.01 |
| Uridine-5-triphosphate·Na | 0.001 | 0.001 |
| Calcium chloride [anhydrous] | 0.2 | 0.2 |
| D-Calcium pantothenate | - | 0.00001 |
| Magnesium sulfate [anhydrous] | 0.09769 | 0.0977 |
| Potassium chloride | 0.4 | 0.4 |
| Sodium acetate [anhydrous] | 0.05 | 0.083 |
| Sodium bicarbonate | 2.2 | 2.2 |
| Sodium chloride | 6.8 | 6.365 |
| Sodium phosphate monobasic [anhydrous] | 0.122 | 0.140 |
| D-Glucose | 6.0 | 4.0 |
| Phenol red·Na | 0.02124 | 0.02 |
| Glutathione | 0.01 | 0.01 |
| D-Glucuronic acid·Na | 0.00388 | 0.0042 |
| Cholesterol | 0.0002 | 0.0002 |
| Tween 80 | 0.005 | 0.005 |
| HEPES | 6.0 | 6.0 |
| Albumin, bovine | 50.0 | 9.37 |
| Neopeptone | - | 3.0 |
| Peptone, special | 5.0 | - |
| Yeast extract | 2.0 | - |
| Gelatin | - | 10.7 |
| **ADDED:** |  |  |
| Rabbit serum | 30 ml/L | 55.1 ml/L |
